# Effect of Strapdown Integration Order and Sampling Rate on IMU-Based Attitude Estimation Accuracy

**DOI:** 10.3390/s18092775

**Published:** 2018-08-23

**Authors:** Jung Keun Lee, Mi Jin Choi

**Affiliations:** Inertial Motion Capture Lab, Department of Mechanical Engineering, Hankyong National University, Anseong 17579, Korea; cmj@hknu.ac.kr

**Keywords:** attitude estimation, IMU, strapdown integration order, sampling rate, Kalman filter

## Abstract

This paper deals with the strapdown integration of attitude estimation Kalman filter (KF) based on inertial measurement unit (IMU) signals. In many low-cost wearable IMU applications, a first-order is selected for strapdown integration, which may degrade attitude estimation performance in high-speed angular motions. The purpose of this research is to provide insights into the effect of the strapdown integration order and sampling rate on the attitude estimation accuracy for low-cost IMU applications. Experimental results showed that the effect of integration order was small when the angular velocity was low and the sampling rate was large. However, as the angular velocity increased and the sampling rate decreased, the effect of integration order increased, i.e., obviously, the third-order KF resulted in better estimations than the first-order KF. When comparing the case where both transient matrix and process noise covariance matrix are applied to the corresponding order and the case where only the transient matrix is applied to the corresponding order but the process noise covariance matrix for the first-order is still used, both cases had almost equivalent estimation accuracy. However, in terms of the calculation cost, the latter case was more economical than the former, particularly for the third-order KF (i.e., the ratio of the former to the latter is 1.22 to 1).

## 1. Introduction

Applications of inertial measurement units (IMUs) consisting of accelerometers and gyroscopes have been exponentially increasing as these units can function as wearable motion sensors because of their sourceless property, i.e., they do not require external location-fixed sources [1,2,3,4,5,6]. Accurate attitude estimation based on an IMU is an important research theme, and therefore, several attitude estimation algorithms have been introduced in literature [7,8,9,10,11,12,13,14,15,16]. In spite of the varieties of the algorithms, the basic concept of estimation is common; that is, gyroscope signals are integrated to predict the attitude, and then accelerometer signals are used to prevent the drift error caused by the error accumulation associated with the integration.

The first prediction step is called strapdown integration as the sensors are rigidly strapped to a body that we want to track. With regard to the strapdown integration of the gyroscope signals, it should be noted that (i) a gyroscope actually provides discrete samples of the angular velocity, rather than continuous signals and (ii) the gyroscope signal is the summation of angular velocity and noise. 

Related to (i), an integration scheme must be used to integrate the sampled signal and the choice of scheme is application-dependent. For short-timespan and low-accuracy applications, a low-order scheme may be sufficient. For more demanding applications, a higher-order scheme may be more appropriate, as discussed in [17]. It is simply natural that the higher-order scheme requires a higher computational cost. Once the integration order is chosen, the remainder of the Taylor series expansion is excluded from the integration equation. This approximation affects estimation accuracy to some extent. Related to (ii), when the gyroscope signals are integrated by an attitude estimation algorithm, errors in the signals propagate through to the calculated attitude. In the case of attitude algorithms using Kalman filters (KFs) which are the most popular cases, the noise-related terms form the process noise covariance matrices.

In literature, high-frequency angular motions—which are of interest in this study—are often discussed with “coning” or “noncommutivity rate” motions [18,19]. Coning motion (in which one or more axes of the system sweeps out a cone in space) is one particular input used to evaluate strapdown inertial navigation systems (INS) and attitude estimation algorithms. As coning is a demanding type of motion, it is admitted that algorithms operating satisfactorily in this environment will satisfy most other requirements [20,21,22,23,24,25]. However, most of these algorithms are in the context of aerospace fields (e.g., for spacecraft attitude determination) and are less related to biomedical or industrial applications that employ low-cost wearable sensors. Therefore, previous research has not investigated the effect of the difference in integration schemes in practical operating environments. The purpose of this research is to provide insights into the effect of the strapdown integration order and sampling rate on the attitude estimation accuracy for low-cost IMU applications.

In this paper, we compare and analyze IMU-based attitude estimation performance according to the strapdown integration order. Based on the attitude estimation KF introduced in [14], first-order, second-order, and third-order KFs are formulated with the first-order, second-order, and third-order strapdown integrations, respectively. Estimation performances of the three different KFs are evaluated under various conditions in terms of angular velocities and sampling rates as the transient matrix of KFs is the function of these two. In addition, we investigate the effectiveness of the exact implementation of the process noise covariance matrix of a KF by comparing the exact implementation to the approximated implementation. Finally, we discuss the estimation accuracy for each case, considering the calculation cost.

## 2. Methods

Measurements from the accelerometer (*A*) and gyroscope (*G*) sensors are respectively modeled as follows:

(1) yA,t=gSt+aSt+nA

(2) yG,t=ωSt+nG,

where gS is the gravity vector, aS is the external acceleration, ωS is angular velocity, $n_{A}$ and $n_{G}$ are the measurement noises of the accelerometer and the gyroscope, respectively, which are assumed to be independent white Gaussian noise with zero mean and covariance matrices, respectively, are $\Sigma_{A}=\sigma_{A}^{2}\;I$ and $\Sigma_{G}=\sigma_{G}^{2}\;I$. The left superscript *S* indicates that the vectors are observed in the sensor frame coordinate. In (1), the external acceleration aS is modeled as a first-order Markov chain stochastic process as in [14], i.e.,

(3) aSt=caaSt−1+cbwat−1

where $c_{a}$ and $c_{b}$ are constant parameters and wat−1 is the corresponding white Gaussian noise with zero mean. Both parameters $c_{a}$ and $c_{b}$ were set to 0.1 in this study.

The attitude estimation KF proposed in [14] predicts the attitude in the time update (prediction step) using the angular velocity measured from the gyroscope and performs the measurement update (correction step) using the gravitational acceleration measured from the accelerometer. This paper is about the prediction step in which the selection of strapdown integration matters.

### 2.1. Strapdown Integration

The discrete-time model for the strapdown integration is [12]

(4) Rt=Rt−1 exp([ωSt−1×]Δt),

where R is the short form of RSI, which is the rotation matrix of the sensor frame *S* with respect to the inertial frame *I*; Δt is the sampling time; [ωS×] represents the vector cross product of ωS=[ωxωyωz]T, i.e.,

(5) [ωS×]=[0−ωzωyωz0−ωx−ωyωx0].

Henceforth, [ωSt−1×]Δt in (4) is simply denoted as $A_{t-1}$ for convenience. 

By the Taylor expansion of the exponential term, (4) can be rewritten as

(6) $$R_{t}=R_{t-1}\;\left(I+A_{t-1}+\frac{A_{t-1}^{2}}{2!}+\frac{A_{t-1}^{3}}{3!}+\cdots \right).$$

Then, by transposing both sides of (6) and applying ${\left[a\times \right]}^{T}=-\left[a\times \right]$, (6) can be rewritten as

(7) $$R_{t}^{T}=\left(I-A_{t-1}+\frac{A_{t-1}^{2}}{2!}-\frac{A_{t-1}^{3}}{3!}+\cdots \right)R_{t-1}^{T}.$$

Because the rotation matrix R contains the three unit column vectors of the inertial coordinate system expressed in the sensor coordinate system, i.e., R=[XSYSZS]T, the specific form of (7) for ZS is

(8) ZSt=(I−At−1+At−122!−At−133!+⋯)ZSt−1

Again, this paper deals with the effect of the strapdown integration order on attitude estimation performance. Accordingly, depending on the strapdown integration order selected, (8) can be written as follows:

(9a) ZS1,t=(I−At−1)ZS1,t−1

(9b) ZS2,t=(I−At−1+At−122!)ZS2,t−1

(9c) ZS3,t=(I−At−1+At−122!−At−133!)ZS3,t−1,

where ZS1,t, ZS2,t, and ZS3,t are the first-order, second-order, and third-order approximations of (8), respectively.

### 2.2. Transient Matrix and Process Noise Covariance Matrix

A linear KF can be defined by the following process and measurement models [14]:

(10) $$x_{t}=\Phi_{t-1}x_{t-1}+w_{t-1}$$

(11) $$z_{t}=Hx_{t}+v_{t},$$

where $x_{t}$ is the state vector, $\Phi_{t-1}$ is the state transition matrix, $w_{t-1}$ is the Gaussian process noise, $z_{t}$ is the measurement vector, H is the observation matrix, and $v_{t}$ is the Gaussian measurement noise. Covariance matrices of $w_{t-1}$ and $v_{t}$ are $Q_{t-1}$ and $M_{t}$, respectively. The state vector is defined as xt=[gStTaStT]T. Note that gS is g×ZS where g is the norm of gravitational acceleration and attitude vector ZS is the sufficient information to calculate the attitude (i.e., roll and pitch) [12]. Therefore, the KF process model is

(12) [gStaSt]=[Φgt−10303caI][gSt−1aSt−1]+[Kgt−100cbI][nGwat−1],

where Φgt−1 and Kgt−1 vary according to strapdown integration orders.

By substituting (2) into (9a–c) and applying $[n_{G}\times ][n_{G}\times ]=0$, the process models in terms of $y_{G,t}$ and $n_{G}$ for the first-order, second-order, and third-order strapdown integrations are as follows, respectively:

(13a) gS1,t=(I−Δt[yG,t−1×])gS1,t−1+Kg1,t−1nG

(13b) gS2,t=(I−Δt[yG,t−1×]+Δt2[yG,t−1×]22!)gS2,t−1+Kg2,t−1nG

(13c) gS3,t=(I−Δt[yG,t−1×]+Δt2[yG,t−1×]22!−Δt3[yG,t−1×]33!)gS3,t−1+Kg3,t−1nG

where

(14a) Kg1,t−1=−Δt[gSt−1×]

(14b) Kg2,t−1=Kg1,t−1+Δt22!([yG,t−1×][gSt−1×]+[([yG,t−1×]gSt−1)×])

(14c) Kg3,t−1=Kg2,t−1−Δt33!([yG,t−1×]2[gSt−1×]+[([yG,t−1×]2gSt−1)×]+[yG,t−1×][([yG,t−1×]gSt−1)×])

From (12), the Φgt−1’s for the first-, second-, and third-order KFs are

(15a) Φg1,t−1=I−Δt[yG,t−1×]

(15b) Φg2,t−1=Φg1,t−1+Δt2[yG,t−1×]22!

(15c) Φg3,t−1=Φg2,t−1−Δt3[yG,t−1×]33!

The process noise covariance matrices for the first-, second-, and third-order KFs are, respectively,

(16a) Q1,t−1=[Kg1,t−100cbI] [ΣG00I] [Kg1,t−100cbI]T

(16b) Q2,t−1=[Kg2,t−100cbI] [ΣG00I] [Kg2,t−100cbI]T

(16c) Q3,t−1=[Kg3,t−100cbI] [ΣG00I] [Kg3,t−100cbI]T

where $Q_{t-1}$ is defined as $E\left[w_{t-1}w_{t-1}^{T}\right]$ and *E* is the expectation operator.

The attitude KF dealt in this paper has the measurement model based on the accelerometer measurement of (1) as follows, regardless of the integration order:

(17) yA,t=[II][gStaSt]+nA,

where the measurement vector $z_{t}$ is $y_{A,t}$, the observation matrix H is $\left[\begin{array}{cc} I & I \end{array}\right]$, the measurement noise $v_{t}$ is $n_{A}$, and the measurement noise covariance matrix $M_{t}$ is $\Sigma_{A}$ [14]. 

### 2.3. Experimental Setup

**Figure 1:**
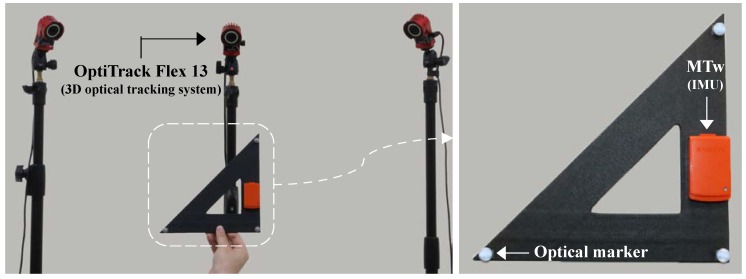
Experiment setup: three optical markers were attached to each vertex of the triangle ruler using double-side adhesive tapes.

For comparing the performance of KFs with respect to the strapdown integration order, an MTw IMU (from Xsens Technologies B.V., Enschede, The Netherlands) was used to provide input data for the aforementioned KFs. In addition, to investigate the estimation accuracy, an OptiTrack Flex13 3D optical tracking system (from NaturalPoint, Inc., Corvallis, OR, USA) was used to obtain the truth reference of the attitude based on the spatial positions of three markers on a plane (see Figure 1).

Because the angular velocity is one of the critical factors causing differences according to the integration order, three different experiments according to angular velocities were carried out. Each experiment repeated 30 times for Monte Carlo analysis. The means ± standard deviations of the averaged $\left\|y_{G}\right\|$ for each experiment are as follows:

- Test A (slow): the average $\left\|y_{G}\right\|$ of nearly 1.47 ± 0.6 rad/s with the average maximum of 6.08 rad/s;
- Test B (fast): the average $\left\|y_{G}\right\|$ of nearly 3.90 ± 0.6 rad/s with the average maximum of 13.70 rad/s;
- Test C (vary fast): the average $\left\|y_{G}\right\|$ of nearly 6.18 ± 0.8 rad/s with the average maximum of 17.16 rad/s.

The IMU measurement signals were delivered by the MTw at a sampling rate of 120 Hz. Then, to investigate the effects of the sampling rate in each test and for each integration order, the 120-Hz data were downsampled to 80-, 40-, and 20-Hz data by interpolation. Finally, a Monte Carlo analysis consisting of the 30 runs was performed.

## 3. Results and Discussions

### 3.1. Case 1: Variation of Transient Matrix and Process Noise Covariance Matrix

Case 1 investigates the effect of the integration order on the estimation accuracy when both the transient matrix shown in (15) and the process noise covariance matrix shown in (16) are varied according to the selected order. In other words, the first-order KF uses Φg1 and $Q_{1}$, the second-order KF uses Φg2 and $Q_{2}$, and the third-order KF uses Φg3 and $Q_{3}$. Table 1 shows the estimation results of roll, pitch, and attitude, in terms of the averaged root-mean-square error (RMSE) from 30 Monte Carlo runs, for different integration orders and for different sampling rates. The attitude error in Table 1 is the angle between the truth reference attitude vector from the optical tracking system (ZSopt) and the estimated attitude vector (ZSest).

In Test A, although the RMSE decreases as the integration order increases, the integration order does not affect the accuracy as much as it does in Test B or C. As the sampling rate decreases, the RMSE increases slightly. In all cases, the RMSE was less than 2.2° for roll and pitch, showing high estimation accuracy.

In Test B in which the angular velocity was higher than in Test A, RMSEs increased in all three KFs compared to those in Test A. For example, the attitude estimation RMSEs of first-, second-, and third-order KFs at 120 Hz were 3.11°, 2.79°, and 2.76°, respectively. More importantly, the estimation performance in Test B was more affected by the selected integration order than that in Test A. The effect of the order on the RMSE was clearer in the lower sampling rate, i.e., the attitude estimation differences between the first-order KF and the third-order KF were 0.35° at 120 Hz and 5.49° at 20 Hz.

In Test C, in which the angular velocity was the highest among the three tests, the RMSEs also increased in all three KFs compared to those in Test B. For example, the attitude estimation RMSEs of first-, second-, and third-order KFs at 120 Hz were 5.26°, 4.26°, and 2.17°, respectively. Note that IMU-based attitude determination under dynamic conditions is a type of “underdetermined” problems because the accelerometer signal used for the correction has two unknowns: the attitude and external acceleration. Therefore, particularly for highly dynamic test conditions like Test C, estimation performance can be seriously degraded even with a high sampling rate such as 120 Hz.

**Figure 2:**
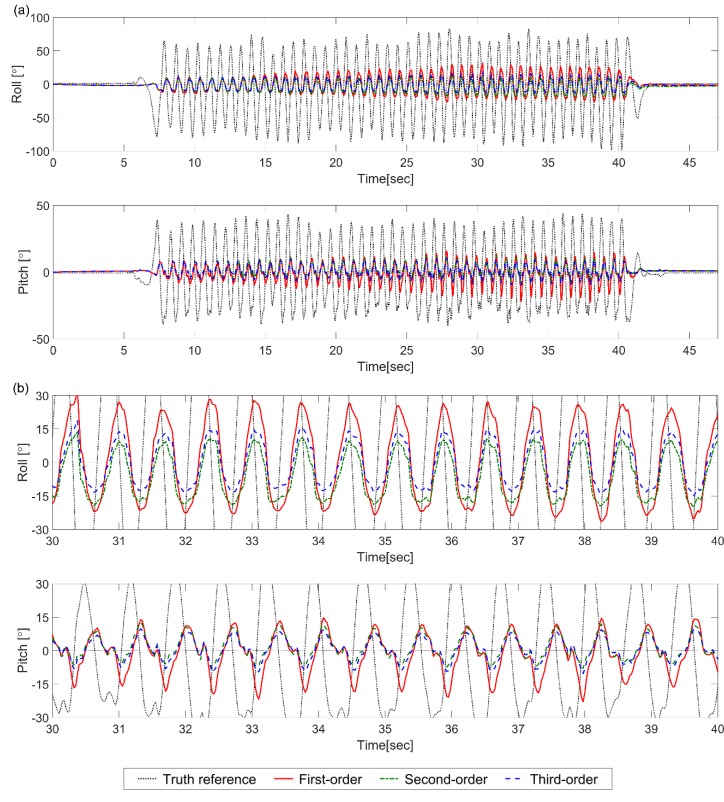
Results of Test C (40 Hz) for Case 1: (**a**) roll and pitch estimation errors from the first-order KF (red solid), the second-order KF (green dot-dash), and the third-order KF (blue dashed) with respect to the truth reference angle (black dotted); (**b**) enlarged subsections for 10 s.

As shown in Figure 2 (from one trial out of 30 runs), in the case of a 40 Hz sampling rate for Test C, estimation errors from the first-order KF were significantly increased as time elapsed, compared to the second-order and third-order KFs. For example, the pitch estimation RMSEs from the first-order, the second-order, and the third-order KFs were 6.92°, 4.22°, and 3.19°, respectively. However, when the sensor returned to the static condition, the estimation accuracy was recovered quickly in all KFs. The difference caused by the integration order increased, as the sampling rate decreased. For example, differences of the pitch estimation RMSEs from the first-order and third-order KFs were 0.75° at 120 Hz, 1.47° at 80 Hz, 3.79° at 40 Hz, and 6.51° at 20 Hz. This tendency can be observed in Test B as well. 

As seen in the three test results, the difference of the attitude estimation performances between the different order KFs became larger as A (=[ωS×]Δt) was larger (i.e., as angular velocity ωS was larger and the time stepsize Δt was larger and thus the sampling rate was smaller). Note that while all the KFs have the same correction procedure, more frequent correction procedures are applied to the cases of higher sampling rate than those of lower sampling rate. This means that the estimation differences shown in Table 1 do not come from the prediction procedure only.

### 3.2. Case 2: Variation of Transient Matrix Only

Case 2 investigates the effect of the integration order on the estimation accuracy when only the transient matrix shown in (15) is varied according to the selected order. In other words, the first-order KF uses Φg1 and $Q_{1}$, the second-order KF uses Φg2 and $Q_{1}$ (which is referred to as second-order”), and the third-order KF uses Φg3 and $Q_{1}$ (which is referred to as third-order”). Table 2 shows the averaged RMSEs from the 30 Monte Carlo runs, for different integration orders and for different sampling rates. Similar to the results of Case 1, the difference of the attitude estimation performances between the different order KFs in Case 2 also became larger as the angular velocity was larger and the sampling rate was smaller. 

Note that in all the three tests, results from the second-order” and third-order” in Case 2 were almost the same as those from the second-order and third-order in Case 1, respectively. This shows that the effect of the process noise covariance matrix according to the integration order on the attitude estimation accuracy is negligible in most cases. It can be observed that some estimation errors from Case 1 were bigger than those from Case 2 (e.g., 9.44° of attitude error from Case 1 versus 9.10° from Case 2, in case of the third-order KF at 20 Hz of Test C). Such results occurred when low sampling rates (i.e., 20 Hz) and fast test conditions (i.e., Tests B and C) were applied and thus estimation accuracies were highly deteriorated.

In terms of computational costs for each approach compared to the first-order KF, the second-order” and third-order” in Case 2 were much more cost-effective than the second-order and third-order in Case 1, respectively, as the latter require the computation of the transient matrix as well as the process noise covariance matrix (see Table 3). In particular, the calculation time of the third-order” is 1.06 times higher than that of the first-order KF. On the other hand, the third-order KF requires 1.29 times more computation time than the first-order KF. However, when we compare the third-order” to the third-order, the former is 1.22 times cost-effective than the latter, whereas the former and the latter have almost the same estimation accuracy. 

Therefore, when we need to choose a higher-order KF to deal with a high angular velocity and a small sampling rate and thus to maintain the estimation accuracy, it is more advantageous to change the transient matrix and to keep the process noise covariance matrix of the first-order integration. 

## 4. Conclusions

The attitude of an IMU relative to the global reference frame can be tracked by integrating the angular velocity signal obtained from the gyroscope in the IMU. In many low-cost and low-end IMU applications, a first-order integration scheme has been selected. With the improvement of the computer processing ability, the restriction on the calculation amount is reduced and the demand for the estimation accuracy is becoming stronger. Therefore, a higher-order integration scheme may be more easily chosen for low-cost applications than before. 

In this paper, we investigated the effect of strapdown integration order and sampling rate on estimation accuracy under different test conditions. Experimental results showed that the effect of integration order was small when the angular velocity was low and the sampling rate was large. However, as the angular velocity increased and the sampling rate decreased, the effect of integration order increased, i.e., obviously, the third-order KF produced better estimations than the first-order KF. When comparing Case 1 (where both the transient matrix and the process noise covariance matrix are applied for the corresponding order) and Case 2 (where only the transient matrix is applied for the corresponding order but the process noise covariance matrix for the first-order is still used), both cases had almost equivalent estimation accuracy. However, in terms of the calculation cost, Case 2 is cheaper or more cost-effective than Case 1, particularly for the third-order (i.e., ratio of Case 1 to Case 2 is 1.22 to 1). Therefore, it can be concluded that Case 2 is superior to Case 1, overall.

## Figures and Tables

**Table 1:** Averaged root-mean-square error (RMSE) results for Case 1 (unit: °).

Sampling Rate (Hz)	Roll	Pitch	Attitude	Roll	Pitch	Attitude	Roll	Pitch	Attitude
**Test A**	**First-order**			**Second-order**			**Third-order**		
120	1.12	0.85	1.60	1.05	0.76	1.47	1.05	0.76	1.47
80	1.17	0.93	1.68	1.07	0.78	1.50	1.07	0.78	1.49
40	1.38	1.23	1.98	1.20	0.95	1.70	1.20	0.94	1.67
20	2.13	2.13	2.93	1.70	1.57	2.41	1.67	1.54	2.31
**Test B**	**First-order**			**Second-order**			**Third-order**		
120	2.50	2.00	3.11	2.27	1.71	2.79	2.26	1.69	2.76
80	2.80	2.29	3.50	2.34	1.82	2.93	2.32	1.75	2.86
40	4.35	3.74	5.49	2.82	2.51	3.82	2.68	2.18	3.42
20	8.48	7.29	10.85	5.19	5.21	7.39	4.12	3.58	5.36
**Test C**	**First-order**			**Second-order**			**Third-order**		
120	4.52	3.23	5.26	3.70	2.57	4.26	3.66	2.48	4.17
80	5.42	4.00	6.42	3.88	2.74	4.52	3.79	2.53	4.33
40	8.87	6.92	10.92	5.18	4.29	6.47	4.46	3.13	5.25
20	16.33	12.61	21.11	11.02	9.78	14.01	7.67	6.10	9.44

**Table 2:** Averaged RMSE results for Case 2 (unit: °).

Sampling Rate (Hz)	Roll	Pitch	Attitude	Roll	Pitch	Attitude	Roll	Pitch	Attitude
**Test A**	**First-order**			**Second-order″**			**Third-order″**		
120	1.12	0.85	1.60	1.05	0.76	1.47	1.05	0.76	1.47
80	1.17	0.93	1.68	1.07	0.79	1.50	1.07	0.78	1.49
40	1.38	1.23	1.98	1.20	0.95	1.70	1.20	0.54	1.67
20	2.13	2.13	2.93	1.70	1.57	2.41	1.67	1.54	2.32
**Test B**	**First-order**			**Second-order″**			**Third-order″**		
120	2.50	2.00	3.11	2.27	1.71	2.79	2.26	1.69	2.76
80	2.80	2.29	3.50	2.34	1.82	2.93	2.32	1.75	2.86
40	4.35	3.74	5.49	2.82	2.49	3.82	2.68	2.18	3.42
20	8.48	7.29	10.85	5.14	5.06	7.24	4.12	3.58	5.39
**Test C**	**First-order**			**Second-order″**			**Third-order″**		
120	4.52	3.23	5.26	3.70	2.57	4.26	3.66	2.48	4.17
80	5.42	4.00	6.42	3.89	2.74	4.53	3.80	2.54	4.34
40	8.87	6.92	10.92	5.17	4.22	6.43	4.46	3.19	5.29
20	16.33	12.61	21.11	11.69	9.98	14.60	7.24	5.93	9.10

**Table 3:** Calculation costs of the higher-order Kalman filters (KFs) with respect to the first-order KF.

KFs	Calculation Cost
First-order KF	1
Second-order KF	1.12
Second-order″ KF	1.04
Third-order KF	1.29
Third-order″ KF	1.06

## References

[1] Sabatini A.M. (2011). Estimating three-dimensional orientation of human body parts by inertial/magnetic sensing. Sensors.

[2] Zizzo G., Ren L. (2017). Position tracking during human walking using an integrated wearable sensing system. Sensors.

[3] Marina H.G., Espinosa F., Santos C. (2012). Adaptive UAV attitude estimation employing unscented Kalman filter, FOAM and low-cost MEMS sensors. Sensors.

[4] Maguire S.T.G., Robertson P.A. (2017). UAV attitude estimation using low-frequency radio polarization measurements. IEEE Trans. Aerosp. Electron. Syst..

[5] Huang W., Fang T., Luo L., Zhao L., Che F. (2017). A damping grid strapdown inertial navigation system based on a Kalman filter for ships in polar regions. Sensors.

[6] Lee J.K., Robinovitch S.N., Park E.J. (2015). Inertial sensing-based pre-impact detection of falls involving near-fall scenarios. IEEE Trans. Neural Syst. Rehab. Eng..

[7] Roetenberg D., Luinge H.J., Baten C.T., Veltink P.H. (2005). Compensation of magnetic disturbances improves inertial and magnetic sensing of human body segment orientation. IEEE Trans. Neural Syst. Rehab. Eng..

[8] Lee J.K., Choi M.J. (2017). A sequential orientation Kalman filter for AHRS limiting effects of magnetic disturbance to heading estimation. J. Electr. Eng. Technol..

[9] Madgwick S., Harrison A., Vaidyanathan R. Estimation of IMU and MARG orientation using a gradient descent algorithm. Proceedings of the 2011 IEEE International Conference on Rehabilitation Robotics (ICORR).

[10] Suh Y.S. (2010). Orientation estimation using a quaternion-based indirect Kalman filter with adaptive estimation of external acceleration. IEEE Trans. Instrum. Meas..

[11] Lee J.K., Park E.J. (2009). A fast quaternion-based orientation optimizer via virtual rotation for human motion tracking. IEEE Trans. Biomed. Eng..

[12] Lee J.K., Park E.J., Robinovitch S.N. (2012). Estimation of attitude and external acceleration using inertial sensor measurement during various dynamic conditions. IEEE Trans. Instrum. Meas..

[13] Lee J.K., Park E.J. (2009). Minimum-order Kalman filter with vector selector for accurate estimation of human body orientation. IEEE Trans. Robot..

[14] Ligorio G., Sabatini A.M. (2015). A novel Kalman filter for human motion tracking with an inertial-based dynamic inclinometer. IEEE Trans. Biomed. Eng..

[15] Feng K., Li J., Zhang X., Shen C., Bi Y., Zheng T., Liu J. (2017). A new quaternion-based Kalman filter for real-time attitude estimation using the two-step geometrically-intuitive correction algorithm. Sensors.

[16] Song M., Wu W., Wang J. (2013). Error analysis of classical strapdown velocity integration algorithms under maneuvers. J. Guid. Control Dyn..

[17] Woodman O.J. (2007). An Introduction to Inertial Navigation; UCAM-CL-TR-696.

[18] Wang M., Wu W., Wang J., Pan X. (2015). High-order attitude compensation in coning and rotation coexisting environment. IEEE Trans. Aerosp. Electron. Syst..

[19] Ignagni M. (1996). Efficient class of optimized coning compensation algorithms. J. Guid. Control Dyn..

[20] Miller R.B. (1983). A new strapdown attitude algorithm. J. Guid. Control Dyn..

[21] Gao W., Zhang Y., Wang J. (2014). A strapdown inertial navigation system/Beidou/Doppler velocity log integrated navigation algorithm based on a cubature Kalman filter. Sensors.

[22] Ahmed M.S., Cuk D.V. (2005). Strapdown attitude algorithms using quaternion transition matrix and random inputs. Sci. Tech. Rev..

[23] Lee J.G., Yoon Y.J. (1990). Extension of strapdown attitude algorithm for high-frequency base motion. J. Guid. Control Dyn..

[24] Bortz J.E. (1971). A new mathematical formulation for strapdown inertial navigation. IEEE Trans. Aerosp. Electron. Syst..

[25] Savage P.G. (1998). Strapdown inertial navigation integration algorithm design. Part 1: Attitude algorithms. J. Guid. Control Dyn..

